# Comparison of Predator-Prey Model and Hawk-Dove Game for Modelling Leukemia

**DOI:** 10.1155/2022/9957514

**Published:** 2022-09-22

**Authors:** Mariam Sultana, Fareeha Sami Khan, M. Khalid, Areej A. Al-moneef, Ali Hasan Ali, Omar Bazighifan

**Affiliations:** ^1^Department of Mathematical Sciences, Federal Urdu University of Arts, Science & Technology, University Road, Gulshan-e-Iqbal Campus, Karachi-75300, Pakistan; ^2^Department of Mathematical Sciences, College of Science, Princess Nourah Bint Abdulrahman University, P.O. Box 84428, Riyadh 11671, Saudi Arabia; ^3^Department of Mathematics, College of Education for Pure Sciences, University of Basrah, Basrah, Iraq; ^4^Doctoral School of Mathematical and Computational Sciences, University of Debrecen, H-4002 Debrecen, Pf. 400, Hungary; ^5^Department of Mathematics, Faculty of Education, Seiyun University, Hadhramout 50512, Yemen

## Abstract

Game theory is an excellent mathematical tool to describe the interaction between the immune system and cancerous leukocytes (*c*.*leu*). The feature of cancerous leukocytes to differentiate and mutate to give rise to leukemia is in the domain of ecological models as well. In this work, the dynamic of leukemia is described and compared by two models: firstly by a simple probabilistic mathematical model using the zero-sum two player game of Hawk and Dove, and secondly by Leslie Predator Prey model of ecology. The main goal of this study is to compare the results of both models and then discuss the treatment of leukemia i.e., Hematopoietic Stem cell transplant with the best model among them. Hawk and Dove model also describes the cell to cell interaction of cancerous leukocytes and healthy leukocytes (*leu*) after diagnoses and the condition of the patient before and after treatments. In this work, Hematopoietic Stem cell transplant is discussed by using concepts of a zero-sum three player game. Also, both models will be characterized by determining the stability properties, identifying basins of attraction, and locating the equilibrium points to see, at what extent the patient's survival is possible with leukemia in its body. Results for both models will be presented graphically.

## 1. Introduction

In blood there are several types of diseases that can occur, among them is “hematological malignancy.” This term refers to the type of malignancy that can affect lymph nodes, bone marrow, and blood as well. Among hematological malignancy, leukemia was discovered in 1847 by Rudolf Virchow. In Leukemia, the production of a certain kind of white blood cells known as “the blasts” increases, and the normal function of blood and bone marrow gets disturbed. Leukemia is mainly categorized into four types: Acute lymphocytic leukemia (ALL), Chronic lymphocytic leukemia (CLL), Acute myelogenous leukemia (AML), and Chronic myelogenous leukemia (CML). Leukemia, up to some extent, is regarded as the treatable disease in the medical world. For this cause, medical and all other branches of science and technology have been continuously trying to find the cure and efficient drugs to tackle this disease in order to relieve the curb and misery of mankind. To examine the root causes and their therapies, there are plenty of approaches. With the available scientifically obtained data from computer aided models, the mathematical and statistical approach has paved the way for new useful medical investigations. Research on Leukemia is underway. Furth & Makhn [1] showed in history, the transmission of leukemia in mice, for the very first time. Later on, many mathematical models and treatments were suggested based on intercellular interactions by [2–4]. A further detailed model demonstrated the interactions of myeloblasts with neutrophils, *T*-cells and leukemia and also provided the treatment model for acute myeloblastic leukemia chemotherapy, see [5]. The model was proposed by Helen & Natasha [6] based on the usual differential equations explaining the interaction of effector *T*-cell, a naive *T*-cell in chronic myelogenous leukemia. Huge changes in the growth and death rates of chronic myelogenous leukemia cells can be observed in the results.

Recently, some authors investigated that the patients prognosis of acute myeloid leukemia is due to cytokine-independent leukemic cell proliferation see [7]. Miguel [8] & Rodriguesa et.al [9], also contributed their results for cancer metabolism and presented a detailed mathematical model for chemoimmunotherapy for lymphocytic leukemia describing its normal conditions and stability analysis of immunotherapy. Leukemia and tumor based on several other mathematical models were studied by a few more researchers such as [10–13]. On the other hand, many other models related to differential equations were studied recently by several authors, see [14–26].

It is important to mention here, that all discussed models were furnished either with differential equations or numerical methods but the use of Game theory for a such complex disease is a recent advancement. Game theory is very effective in describing the interaction between the immune system and cancer leukocytes. Whereas the interrelation of stem cell after the transplant with leukocytes against cancerous leukocytes or with cancerous leukocytes against leukocytes is in the domain of evolutionary game theory.

In this century, a new arising treatment of leukemia i.e., stem-cell transplantation has gained the interest of researchers of different fields. By the hematopoietic stem-cell idea, Pappenheim [27] initiated stem-cell biology. Since they are capable of differentiating into the main three major life cells, but also replicating into another cell of their own kind, until the end of life, the process of differentiation of the same *DNA* continues. This treatment was first performed by Main & Prehn [28] in the mice. Stem-cells have a history that is very fascinating. The researchers observed after the discovery of stem-cells that leukemia tumours have the ability to develop in vitro and vivo colonies, see [29]. This finding and other postulates led to the result that, just as other normal cells differentiate, stem-cells have the capacity to indefinitely differentiate themselves. Cancer stem-cells were first investigated by Lapidot et.al [30] in acute myeloid leukemia and Cobaleda et.al [31] for acute lymphocytic leukemia. These studies captured the interest of mathematicians so many mathematical models have been developed for the treatment of different diseases. Boni et.al [32] suggested a model using evolutionary game theory for the interaction of stem-cells in bioactive scaffolds (i.e., cardiac muscles that lose elasticity). Mneimneh et. al [33] described a game-theoretic and stochastic survivability mechanism against induced attacks in cognitive radio networks. Torkaman et. al [34, 35] developed a system for classifying leukemia based on a cooperative game based on the population of Iran.

With all the available treatments yet, a leukemia patient can not be treated completely but his survival is possible with long term treatments such as chemotherapy, immunotherapy, radiation, and stem cell transplantation. But in all these treatments the major drawback is that these medicines while targeting the *c*.*leu*destroy the immune system of the patient too. It is yet a challenge to find a such cure that can only target the cancerous cells and improve the life quality of such patients by lessening their pain and suffering. Therefore, this paper is very important in understanding the cell-cell interaction for medical science to gain capabilities of improving the diagnostic techniques and improve survival rate and quality of medicines.

**Figure 1:**
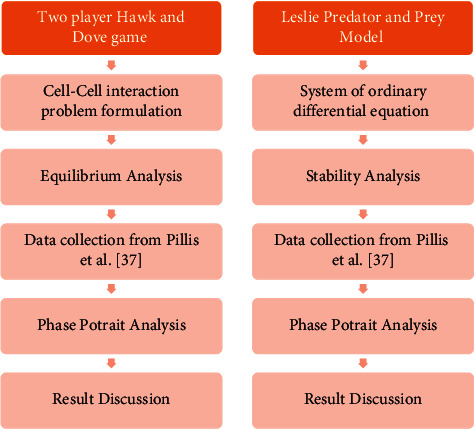
Flow chart of the methodology for mathematical modeling of leukemia.

**Figure 2:**
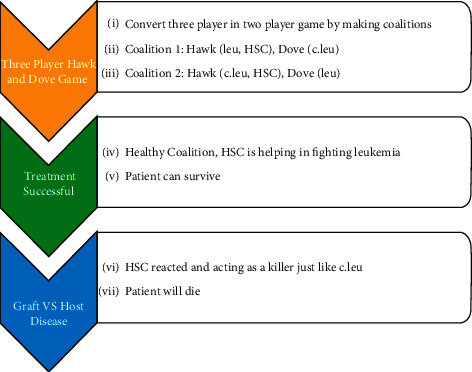
Flow chart of methodology for mathematical modeling of treatment of leukemia.

This paper combines the evolutionary game theory and ecology, predator-prey model with life threatening disease i.e., leukemia. The main goal of this study is to apply replicator dynamics and the Leslie predator-prey model to the dynamics of leukemia see Figure 1. Then, treatment of leukemia i.e., stem cell transplant is discussed with the concept of evolutionary game theory because it is easier to formulate the cell to cell interaction by this methodology instead of the Leslie Predator Prey model see Figure 2.

In this paper, significant contributions have been made in both directions: (1) in terms of the formal analysis of this disease, this work has focused on the proposed extension model of an existing formalism. The viability and effectiveness of this proposal are corroborated by the application of both formalism to the analysis. (2) With regard to the design of leukemia dynamics, this approach is based on applying population dynamics, and to generate a mathematical model which can be proven rationally within an underlying game theoretical framework.In this paper, Section 2 is about showing leukemia in a fast time scale using the replicator dynamics model.  Section 3 describes the leukemia Dynamics by the Predator Prey model.  Section 4 is about a patient's condition after stem-cell transplant therapy in view of replicator dynamics.  Section 5 discusses the results of replicator dynamics for Hematopoietic Stem-Cell Transplantation. Also, discusses the numerical values of parameters (i.e cell count of each cell type) among calculations to show the graphical illustrations.  Section 6 is the discussion of the results. In Section 7, Conclusion and Future Work is given.

## 2. Leukemia Dynamics by Hawk and Dove Game

The principles of game theory provide a common framework for creating structures and, eventually, comprehending several significant cancer problems. A game can be defined as a set of comparisons and the opportunity to play the game in line with the game's rules. The classical game theory is founded on a set of strictly defined axioms about game structures. It is assumed in game theory that each player or agent has predefined goals, choices, and interests, which are described by a function called the “utility function.” Again, utility refers to the most gains that players may receive as an outcome of the game, and each player's goal, theoretically, is to yield maximum profit or value, theoretically.

- Their ethical and viable plan of action in view of the rules.
- The outcome, that players are getting, represent every move they could possibly make given the vast array of strategies at their disposal.

**Figure 3:**
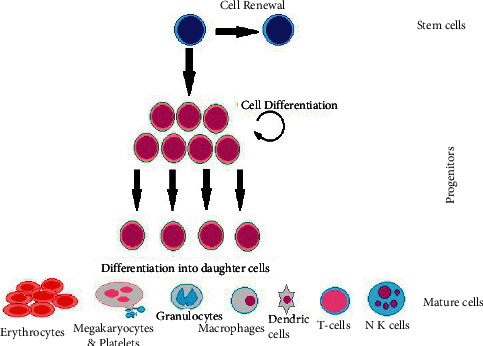
Stem-cell proliferation showing the ability of self renewal and replication into mature cells. This is an infinite process and stem-cells are the only ones who can replicate themselves many times unlike other cells. Unusual proliferation or accelerated cell division can cause leukemia or tumours.

Since acute or chronic leukemia are just different time scale cases theoretically, a case of acute leukemia is taken in consideration in this research (see [36]). Leukocytes, or white blood cells, are a part of the immune system that protect the body from diseases or external invaders (see Figure 3).

While in leukemia, some of these healthy leukocytes get defected and works against the host body and do not let the healthy cells work either. That is the point when tumours are formed called neoplasm. Two types of replicating cells are investigated in this work i.e., cancerous leukocytes (c.leu) and normal leukocytes cells (leu). There will be lower but equal probabilities for *leu* to further multiply into large numbers, resulting in the ability to work as *leu*. In the process, the capacity to sustain neoplastic growth is acquired mainly by those cells which enjoy the capacity for self-renewal. This model concludes that only a limited and restricted number of cells inside the tumour are expected to relaunch tumour development. As per the stochastic theory, the tumour cells are relatively homogeneous, all of these tumour cells undergo active genetic modifications and progress towards malignancy growth. The replicator model suggests that the biologically and functionally explicit population of tumour-launching cells is limited. Therefore the focus of research is on those *leu* breeds that support the progression of targeted treatment for leukemia and this will strongly prevent disease recurrence. Simple and incomplex hypotheses have been made, for the initial discussion of the replicator dynamics model in terms of leukemia. But for more detailed analysis, more and more complex variables that can simulate the reality will be used in the future model.A set of pay off matrix in the evolutionary game theory, i.e., sums, costs, and benefits, of a specific strategy are calculated in terms of success and failure of *leu* and *c*.*leu*. To begin with the test, let the specific (*c*.*leu*, *leu*) phenotypes be set as external stimuli, in the general context of the payoff matrix.

(1) $$\begin{array}{c} A=\left[\begin{array}{cc} a_{11} & a_{12}\\ a_{21} & a_{22} \end{array}\right]\text{.} \end{array}$$

 Here, *a*_*ij*_ is the player's payoff using the *i* plan as opposed to the *j* plan player, since the matrix occupancy acquired in the form as seen in Table 1 is applied in this case, assuming that the interaction between the same type of cells stays neutral, which is why only diagonals are zeros. On the other hand, the *leu*-*c*.*leu* interplay capitulates into a negative payoff.

In particular, for evolutionary game theory, this scenario exactly resembles the Hawk and Dove game, where *leu* denotes the Doves and *c*.*leu* denotes the Hawks. Let the total number of cells be denoted by *p* in time *t* and this further can be classified into two types *c*.*leu* are *P*_*L*_(*t*) and *leu* are *P*_*I*_(*t*). The cell masses selecting *S*_*i*_ strategy i.e., *S*_*i*_={*s*_1_, *s*_2_,…, *s*_*k*_} where *i*=1,2 at *t*. Let *x*_1_′(*t*) and *x*_2_′(*t*) denote the cells proportion of Hawk and Dove strategies, respectively.

(2) $$\begin{array}{c} x_{1}^{\prime }\left(t\right)=\frac{P_{L}\left(t\right)}{p\left(t\right)}\text{,}\\ x_{2}^{\prime }\left(t\right)=\frac{P_{I}\left(t\right)}{p\left(t\right)}\\ =1-x_{1}^{\prime }\left(t\right)\text{.} \end{array}$$

The normalized symmetric matrix in Table 1 gives the general replicator dynamics equation as follows:

(3) $$\begin{array}{c} x_{1}^{\prime }\left(t\right)=\left(ax_{1}-bx_{2}\right)x_{1}x_{2}\text{,}\\ x_{2}^{\prime }\left(t\right)=\left(bx_{2}-ax_{1}\right)x_{1}x_{2}\text{.} \end{array}$$

Fitness is referred as the payoff in replicator dynamics. *H*_*i*_ is the fitness of the *i* phenotype, represented in terms of the *A* fitness and payoff matrix phenotypes, where the cell population's fitness function is *H*_*i*_=(*Ax*)*i* and $\bar{H}$ is the average fitness of the population of cells i.e., $\bar{H}=\sum_{i}{x_{i}H}_{i}=x^{T}Ax$. Taking *x*1+*x*2=1 and with some algebra, the growth rate of *c*.*leu* and *leu* by selecting a strategy is *Si* : *x*(1 − *x*)(*H*1 − *H*2) at any time *t*, here *x* is the frequency of cells. There are the three stationary points i.e., 0,1, and *d* frac *H*1*H*2=*x∗*. For *H*_1_ < *H*_2_ it can be stated that *x*^*∗*^ is dimorphic. Also, for (1 − (*H*_1_/*H*_2_))*leu* and (*H*_1_/*H*_2_)*c*.*leu*, population cells is the polymorphic and stationary point is asymptotically stable. If *H*_1_ > *H*_2_, which means *x*^*∗*^=1 then the stationary point is asymptotically stable, which means there are only *c*.*leu*.

**Figure 4:**
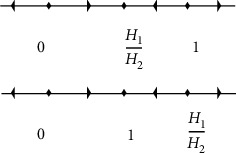
For *n*=2, the replicator dynamic phase plane showing the three equilibrium points as 0, 1 and a dimorphic equilibrium *x*^*∗*^.

In Figure 4 the unique symmetric mixed Nash equilibrium is asymptotically stable i.e., (*H*_1_/*H*_2_). Also, both pure Nash equilibrium of *leu* and *c*.*leu* are asymptotically stable. The risk-dominant one has the larger basin of attraction which is true in general because *x*^*∗*^ > (1/2) for games with an efficient equilibrium and a risk dominant one.

In this paper, the dynamical behaviour of the cells in mathematical models, is illustrated with the help of Mathematical software. These portraits are within the standard ranges of *leu* and *c*.*leu* blood samples of patients recently diagnosed with leukemia, see [37]. In leukemia, *leu* counts differ rarely or sometimes at regular intervals ranging from 30 to 200 × 10^9^ cells/liter. This variance was recorded during the 40 to 80 days time frame, which is a significantly long time relative to the *c*.*leu* life span and maturation acquisition.

**Figure 5:**
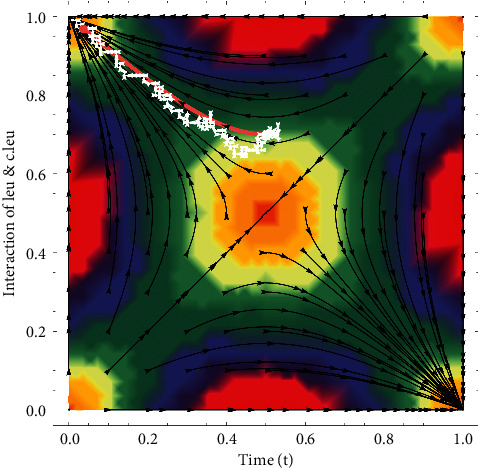
Diagnosis of leukemia is shown through the replicator dynamic phase plane.

**Figure 6:**
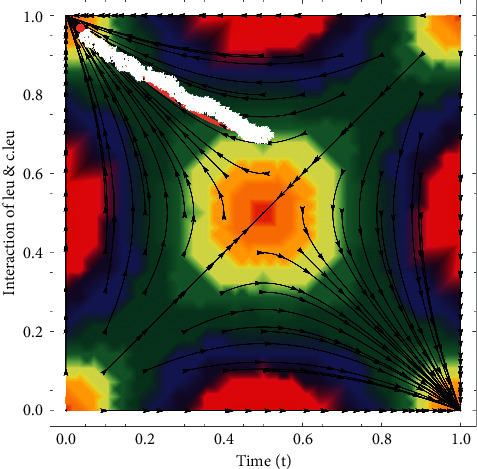
*leu* and *c*.*leu* phase plane of interaction. At the time of the spread of the disease when the patient is near death.

In these portraits, the white zigzag trajectory shows *c*.*leu* and pink dotted line shows *leu*. In Figure 5*c*.*leu* is greater in number than *leu* and if not treated soon *c*.*leu* will make *leu* extinct and the patient will die with the passage of time. Also, the trajectory shows an unstable node as the direction is moving away from the point. *c*.*leu* is much larger in amount than *leu* in Figure 6 and with the passing time, *c*.*leu* makes *leu* extinct and the patient dies. As the path shifts away from the point, the trajectory also shows unstable nodes. So this is the point where the cell population is monomorphic, which is that there will be just *c*.*leu*. It is studied by using fast-scale techniques that if not treated, how quickly a patient will die of acute leukemia.

## 3. Leukemia Dynamics by Leslie Predator Prey Model

The Leslie Predator-Prey Model has a significant impact on the field of mathematical biology and ecology. If the cancerous leukocytes (predator) density is *l*(*t*) and the leukocytes (prey) density is *i*(*t*), then Leslie's model can be represented by systems of nonlinear differential equations as follows:

(4) $$\begin{array}{c} \frac{di}{dt}=\left(r_{1}-c_{1}l-bi\right)i\text{,} \end{array}$$

(5) $$\begin{array}{c} \frac{dl}{dt}=\left(r_{2}-c_{2}\frac{l}{i}\right)l\text{.} \end{array}$$

In equation (5), the factor *c*2(*l*/*i*) is defined as Gower Term. The reason for adding the Gower word comes from the leukocyte growth rate factor, as countless (*i*⟶*∞*) rise relative to the per capita growth of the cancerous leukocytes reaches their maximum growth i.e., ((1/*l*)(d*l*/d*t*)⟶*r*_2_). On the other hand, when leukocyte population declines i.e., (*i*⟶0), then (1/*l*)(d*l*/d*t*)⟶−*∞* (i.e., cancerous leukocytes must vanish). Both cells have logistic growth. *r*_1_ is the growth rate of *leu* with carrying capacity (*r*_1_/*b*_1_) and *r*_2_ is the growth rate of *c*.*leu* with carrying capacity (*c*_2_/*i*) in proportion to the population size of leukocytes. *c*_1_ and *c*_2_ provides the estimate of the quantity of transformation of *c*.*leu* and *leu* into each other respectively. The constant *b* signifies the effectiveness of *c*.*leu* in eliminating *leu*. In Leslie's model it was suggested that the carrying capacity of a predator should be equal to prey. Also, it should be emphasized that the upper limits associated with predator prey together are met under the following conditions:

- For Predators: when there are more predators than prey in the population ratio
- For Prey: when the ratio of Prey to Prey population is high

To estimate the level of success of the design function for achieving the set targets, take into consideration another type of function that acts as a fitness function and intends to represent the net outcome as a single merit value as follows:

(6) $$\begin{array}{c} H_{1}=\frac{r_{1}}{k_{1}}\left(k_{1}-i\right)-bl\text{,} \end{array}$$

(7) $$\begin{array}{c} H_{2}=r_{2}\left(1-\frac{l}{cbi}\right)\text{,} \end{array}$$

where *H*_1_ and *H*_2_ are the fitness functions of *leu* and *c*.*leu*, respectively. For a detailed derivation of equation (6) please see [36]. In contrast with *c*.*leu*, equation (7) defines the increased death rate of *leu*. *c* is the nutritional value constant of *leu*, which is proportional to *c*.*leu*. The densities of *leu* and *c*.*leu* at *t* time are *i* and *l*. Also, *r*_1_ & *r*_2_ are the intrinsic growth rate of *leu* and *c*.*leu*, *k* is the carrying capacity. The assumptions of this model are that a predator is intruding into each other's behavior. This intervention has a negative effect on the predators themselves and increases the mechanism to bring the dramatic shifts in equilibrium, stability, and strength in the population of *c*.*leu* and *leu*. Consider the difference equation of equations (6) & (7) to be interpreted to understand this phenomenon more closely,

(8) $$\begin{array}{c} i\left(t+1\right)=i\left(1+\frac{r_{1}}{k_{1}}\left(k_{1}-i\right)-bl\right)\text{,}\\ l\left(t+1\right)=l\left(1+r_{2}\left(1-\frac{l}{cbi}\right)\right)\text{.} \end{array}$$

Solving equation (5) the following equilibrium point is obtained *E*=*r*_1_*c*_2_/(*bc*_2_+*c*_1_*r*_2_), *r*_1_*r*_2_/(*bc*_2_+*c*_1_*r*_2_). The Jacobian matrix of equation (5) at this equilibrium point is given by the following equation:

(9) $$\begin{array}{c} J_{\left(i,l\right)}=\left[\begin{array}{cc} \frac{-r_{1}bc_{2}}{bc_{2}+c_{1}r_{2}} & \frac{-r_{1}c_{1}c_{2}}{bc_{2}+c_{1}r_{2}}\\ \frac{r_{2}^{2}}{c_{2}} & -r_{2} \end{array}\right]\text{.} \end{array}$$

Therefore the characteristic equation is

(10) $$\begin{array}{c} \lambda^{2}+\frac{r_{1}bc_{2}+\left(bc_{2}+c_{1}r_{2}\right)r_{2}\lambda }{bc_{2}+c_{1}r_{2}}+\frac{r_{1}br_{2}c_{2}+r_{1}c_{1}r_{2}^{2}}{bc_{2}+c_{1}r_{2}}=0\text{.} \end{array}$$

Obviously, *λ*_1_*λ*_2_=(*r*_1_*br*_2_*c*_2_+*r*_1_*c*_1_*r*_2_^2^/*bc*_2_+*c*_1_*r*_2_) > 0 and *λ*_1_+*λ*_2_=−(−*r*_1_*bc*_2_+(*bc*_2_+*c*_1_*r*_2_)/*bc*_2_+*c*_1_*r*_2_) < 0. Hence equilibrium *E*=(*r*_1_*c*_2_/(*bc*_2_+*c*_1_*r*_2_)), (*r*_1_*r*_2_/(*bc*_2_+*c*_1_*r*_2_)) is an asymptotically stable node if ${\left(\sqrt{r_{1}}-\sqrt{r_{2}}\right)}^{2}\geq \left(r_{1}c_{1}r_{2}/bc_{2}+c_{1}r_{2}\right)$ or focus if ${\left(\sqrt{r_{1}}-\sqrt{r_{2}}\right)}^{2}<\left(r_{1}c_{1}r_{2}/\left(bc_{2}+c_{1}r_{2}\right)\right)$. Also, the positive equilibrium (*x*^*∗*^, *y*^*∗*^) of system equation (5) is globally stable, where *x*^*∗*^=(*r*_1_*c*_2_/(*bc*_2_+*c*_1_*r*_2_)) & *y*^*∗*^=(*r*_1_*r*_2_/(*bc*_2_+*c*_1_*r*_2_)).

**Figure 7:**
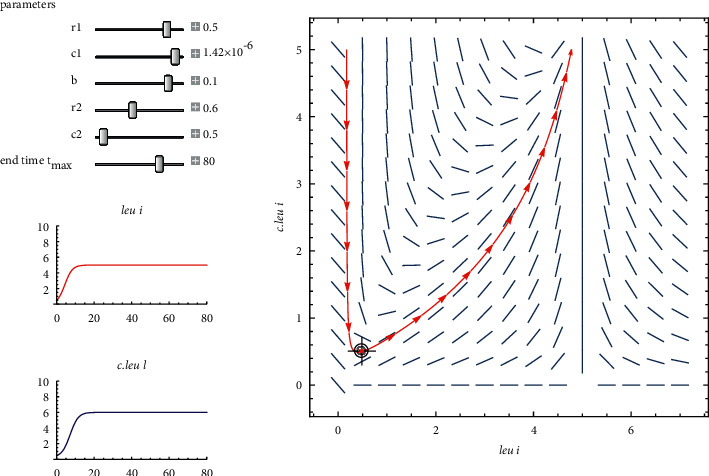
A phase plane of a two-dimensional Leslie predator-prey model that demonstrates the cohabitation of *leu* & *c*.*leu*.

Parameters, i.e., modest intraspecific competition, are shown on the left side of Figure 7. Leukemia was the patient's original diagnosis. As can be seen in the graph on the left, *leu* and *c*.*leu* coexist in the body. The stable node is thus present during this time interval.

An interspecific competition with high saddles. The parameters are displayed on the left. As an example, acute leukemia *leu*has a typical range of 0 to 6; however, when acute leukemia flares up, the graph shows that *c*.*leu*climbs to 6 or higher and *leu*becomes scarce. This node is unstable.

**Figure 8:**
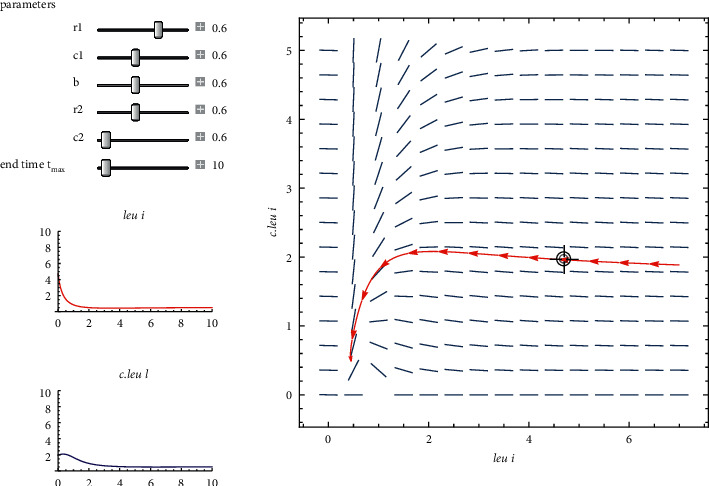
Phase plane for a two-dimensional Leslie predator-prey model.

Figure 8 illustrates how chronic leukemia, or *leu* and *c*.*leu*, can coexist in a patient's body for a very long time. The parameters are displayed on the left. While there is an equilibrium point between *x* = 0 to 2 and *y* = 0 to 4, it is a globally unstable node because the trajectory is moving away gradually. As mentioned above, there are three symptoms that a person with leukemia could encounter. They are all globally stable nodes since all of the estimated critical points are positive.

Typically, a blood sample is taken when a patient has no symptoms yet the disease is present in his blood. Similar to the previous portraits, they are unstable nodes since both healthy and cancerous leukocytes are present in the blood, and in this model, the equilibrium stable point or node occurs when one cell population prevails or survives with a high rate. By utilizing a few algebraic procedures and the mathematical software Mathematica, two different scale models of various areas have been developed and studied. Although different results were found, the scenario remained the same. So let's talk about leukemia treatment in the section afterwards.

## 4. Hematopoietic Stem-Cell Transplantation (HSC)

In this section, a speculative patient who has already been given high chemotherapy measurements and has been transplanted with *HSC*. Since this is a mathematical study and a hypothetical patient is considered here (assumed parameter values are closer to the real patients data described in Section 5.1) so it is important to clarify here that the sole purpose of this study is to show the applicability of a powerful model such as “replicator dynamics” on a complex disease such as “leukemia.” Hence the complications occurring due to chemotherapy and its medicine have not been included in this study. Only the after effects of chemotherapy on blood count is considered in this work.

To understand the interaction of *HSC*, *leu*, and *c*.*leu* after the transplant, game theory have been used. Here are three players, namely, *HSC*, *c*.*leu*, and *leu*, with two execution strategies: kill or not kill. In such a case, there are two cell reaction possibilities when *HSC* enters the body of the patient. One is that, with them, *leu* will react and produce graft versus host disease that is not favourable for the patient and therefore can be said to be grouped with *c*.*leu*. And secondly, to boost the patient's immune system, *HSC* creates a group of *leu* to help destroy the remaining *c*.*leu* to support him in recover.

There is one and only one coalition that could conceivably occur in two-individual games, particularly the coalition between the two players. The amount of possibilities is, obviously greater in games with more than two players, called *n*−individual games. As with this case, the following segments of the players in coalitions could occur in a game with three players:

- For any player on his own
- A coalition between Player 3 and Players 1 & 2
- Players 1 & 3 versus 2
- Players 2 & 3 versus 1
- the grand alliance, all three players included, see [36]

So on the basis of the *HSC* decision, this case has two clear coalitions to decide if it applies to *leu* to fight against the remaining *c*.*leu* or to collate with *c*.*leu* against *leu* i.e.,

- Favourable (*HSC*, *leu*) unfavourable (*c*.*leu*)
- Favourable (*leu*) unfavourable (*c*.*leu*, *HSC*)

It is presumed that interactions between cells of the same types are neutral, therefore, there are zeros on the diagonal, see Tables 2 and 3*). In the presence of the right stimuli that allow the cell to differentiate, while interacting with an unfavourable team,* the favourable team receives a positive payoff, while the unfavourable team receives a negative payoff. This implies not only that it is believed that the favourable team gets a greater payoff under the favourable environment, but also that an unfavourable team will get negative feedback under this environment, see Tables 2 and 3. In Tables 2 and 3 normalized matrices, *F*_1_ stands for a favourable team and *F*_2_ for an unfavourable team.

## 5. Replicator Dynamics and Nash Equilibrium of *HSC* Transplantation

If there are *n* distinct behaviour patterns called “Pure Strategies,” *E*_1_, *E*_2_,…, *E*_*n*_ where *n*=1,2. The state of a population of cells concerning this conflict is fully described by a vector *x*_*i*_=[*x*_1_, *x*_2_,…, *x*_*n*_] ∈ *R*^*n*^, where *x*_*i*_ represents the frequency of *leu* and *c*.*leu* with behaviour *E*_*i*_, *i*=1,2,…, *n*. Consequently, *x*_*i*_ ≥ 0 for all *i* and ∑_*i*_*x*_*i*_=1 the set of all such *x*′*s* is denoted by *S*^*n*^. If an *E*_*i*_ individual cell contends with an *E*_*j*_ individual cell then the payoff will be *a*_*ij*_. Let *A* be the matrix of all those *a*_*ij*_'s. Then, assuming random encounters of cells as,

(11) $$\begin{array}{c} e_{i}.Ax=\underset{i}{\sum }a_{ij}x_{j}\text{,} \end{array}$$

is the average payoff for an *E*_*i*_-cell in a cell population *x* and

(12) $$\begin{array}{c} x.Ax=\underset{i}{\sum }{x_{i}e}_{i}.Ax\\ =\underset{i}{\sum }\underset{j}{\sum }a_{ij}x_{i}x_{j}\text{,} \end{array}$$

is the mean average payoff within cell population *x*. Taylor and Jonker [38] assumed that the growth rates $\left(\dot{x_{1}}/x_{i}\right)=\left(\left(dx_{i}/dt\right)/x_{i}\right)$ of the frequency of strategy *E*_*i*_ are equal to the payoff difference *e*_*i*_.*Ax* − *x*.*Ax*, This yields the replicator equation

(13) $$\begin{array}{c} {\dot{x}}_{1}=x_{i}\left[e_{i}.Ax-x.Ax\right];\;i=1,2,\ldots ,n, \end{array}$$

on the invariant set *S*^*n*^. If the game begins with *x*_*i*_(*t*_°_)=0, so for all *t*, the strategy is *x*_*i*_(*t*)=0. And if there are no favourable cells or unfavourable cells that play strategy from the beginning, then there are no cells that can replicate that approach. The general replicator dynamics equation for normalized symmetric matrix from Tables 2 and 3 is

(14) $$\begin{array}{c} x_{1}^{\prime }=\left(a_{1}x_{1}-a_{2}x_{2}\right)x_{1}x_{2}\text{,}\\ x_{2}^{\prime }=\left(a_{2}x_{2}-a_{1}x_{1}\right)x_{1}x_{2}\text{.} \end{array}$$

After some algebra, these equations become

(15) $$\begin{array}{c} x_{1}^{\prime }=x_{1}\left(1-x_{1}\right)\left(a_{1}-\left(a_{1}+a_{2}\right)x_{2}\right)\text{.} \end{array}$$

Also,

(16) $$\begin{array}{c} x_{1}^{\prime }=\left(b_{1}x_{1}-b_{2}x_{2}\right)x_{1}x_{2}\text{,}\\ x_{2}^{\prime }=\left(b_{2}x_{2}-b_{1}x_{1}\right)x_{1}x_{2}\text{.} \end{array}$$

After some algebra these equations become

(17) $$\begin{array}{c} x_{2}^{\prime }=x_{2}\left(1-x_{2}\right)\left(b_{1}-\left(b_{1}+b_{2}\right)x_{1}\right)\text{.} \end{array}$$

 Which gives five solutions for equations (15) and (17) i.e., the pure strategy unstable solutions are (0,0), (1,1), (1,0), (0,1) or a mixed strategy solution ((*a*_1_/*a*_1_+*a*_2_), (*b*_1_/*b*_1_+*b*_2_)). Clearly, system has positive equilibrium hence the Jacobian matrix at equilibrium is given by the following equation:

(18) $$\begin{array}{c} J^{*}=\left[\begin{array}{cc} \left(1-2x_{1}^{*}\right)\times \left(a_{1}-\left(a_{1}+a_{2}\right)x_{2}^{*}\right) & -x_{1}^{*}\left(1-x_{1}^{*}\right)\left(a_{1}+a_{2}\right)\\ -x_{2}^{*}\left(1-x_{2}^{*}\right)\left(b_{1}+b_{2}\right) & \left(1-2x_{2}^{*}\right)-\left(b_{1}-\left(b_{1}+b_{2}\right)x_{1}^{*}\right) \end{array}\right]\text{.} \end{array}$$

For easier calculation let *a*_1_=*a*, *b*_1_=*b*, (*a*_1_+*a*_2_)=*m*, (*b*_1_+*b*_2_)=*n* then a mixed strategy equilibrium occurs when 0 < (*a*/*m*), (*b*/*n*) < 1 where *x*_1_^*∗*^=(*b*/*n*), *x*_2_^*∗*^=(*a*/*m*). From this, it can be deduced that this system has a positive equilibrium. Therefore, its Jacobian matrix at this equilibrium point becomes

(19) $$\begin{array}{c} J^{*}=\left[\begin{array}{cc} 0 & \frac{-bm}{n}\left(1-\frac{b}{n}\right)\\ \frac{-an}{m}\left(1-\frac{a}{m}\right) & 0 \end{array}\right]\text{.} \end{array}$$

Now, further this matrix describes two cases:

(i) if *a*_1_ & *b*_1_ have different sign, then after solving equations (15) & (17), it is observed that the function shows closed spirals and is monotonic if the following conditions are satisfied:
  - *a*_1_ ≤ (*a*_1_+*a*_2_) & *b*_1_ > (*b*_1_+*b*_2_)
  - *a*_1_ > (*a*_1_+*a*_2_)
(ii) if *a*_1_ and *b*_1_ have same sign than it is a saddle point which means an unstable point. Also, its equilibrium is hyperbolic i.e., (*x*_1_^*∗*^, *x*_2_^*∗*^), so from this, it can be observed that one monomorphic fixed point is asymptotically stable whereas the fixed points under replicator dynamics is a saddle.

### 5.1. Complete Description of Leukocytes (*NK* Cells, Peripheral *CD*34+cells):

The number of *Nk* cells after chemotherapy is given in parameters see [37] where 2.08 × 10^−7^ *Nk* cells and 5.00 × 10^8^ *leu* are remaining cells in the body. The mean value of the peripheral *CD*34+ cells infused was 9.19 × 10^6^ cells/*kg* recipient's body weight and the mean *CD*34+ cell number was 3.47 × 10^4^ cells/*kg*. Whereas Pillis et.al [37] shows remaining *c*.*leu* i.e., 4.31 × 10^−1^ and 1.42 × 10^−6^ cells/*kg*.

**Figure 9:**
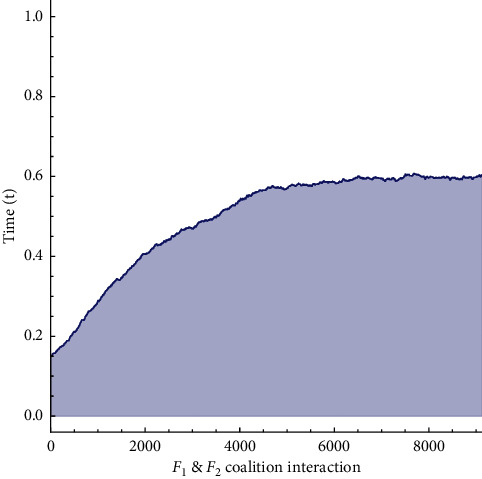
Phase portrait of replicator dynamics after stem-cell transplantation for (*leu*) and (*HSC*, *c*.*leu*).

Figure 9 shows how badly this coalition can affect the patient's life as at the time of chemotherapy the number of *c*.*leu* was minor but after transplantation if graft versus host disease occurs, it can kill the patient. The rise can be observed in an unfavourable coalition of cells. The payoff matrix for this simulation becomes

(20) $$\begin{array}{c} J^{*}=\left[\begin{array}{cc} 0 & 5\times 10^{8}\\ 9.2247\times 10^{6} & 0 \end{array}\right]\text{.} \end{array}$$

**Figure 10:**
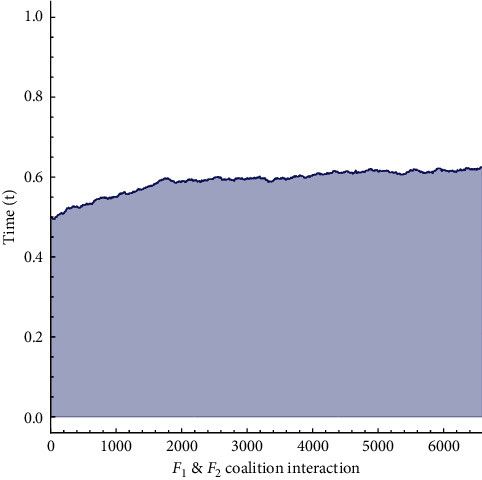
Phase portrait of replicator dynamics after stem-cell transplantation for favourable (*HSC*, *leu*) and unfavourable (*c*.*leu*).

Interaction of favourable and unfavourable coalition of *leu* and *c*.*leu* with *HSC* is described in Figure 10. As it can be seen after a small oscillation the unfavourable group has stopped rising, whereas *leu* is approaching infinity. Payoff matrix for this simulation becomes

(21) $$\begin{array}{c} J^{*}=\left[\begin{array}{cc} 0 & 1009224700\\ 0.431001 & 0 \end{array}\right]\text{.} \end{array}$$

Equations (20). And (21). Gives real and distinct eigen values as they have opposite signs. Equation (20). Have de t(*J*^*∗*^)=−4.61235 × 10^15^ < 0, *τ*^2^ − 4 de t(*J*^*∗*^) > 0 shows a saddle point. Equation (21). Also, have distinct and real eigen values and de t(*J*^*∗*^)=−434976854.9 < 0, *τ*^2^ − 4 de t(*J*^*∗*^)=83424.39498 > 0 which means its also a saddle point.

## 6. Discussion of Results

According to the calculations, it showed that replicator dynamics has three stationary points, 0,1 and (*H*_1_/*H*_2_)=*x*^*∗*^. Also, it showed that *x*^*∗*^ is dimorphic for *H*_1_ ≤ *H*_2_ which means that in blood, both cell populations (i.e., *leu*and c.leu) exist.

For asymptotically stable stationary point, it showed that the cell population is polymorphic i.e., 1 − (*H*_1_/*H*_2_)*c*.*leu*and (*H*_1_/*H*_2_) leu.

For monomorphic cell population i.e., only *c*.*leu*exists, where *x*^*∗*^=1, for *H*_1_ ≥ *H*_2_ which implies that *x*^*∗*^ is asymptotically stable.

By considering the data of a patient who has been diagnosed with leukemia, the two-dimensional Leslie predator prey model of equation (10). Shows in Figure 7 a stable node, that is, ${\left(\sqrt{r_{1}}-\sqrt{r_{2}}\right)}^{2}=4.5548\times 10^{-3}$ and (*r*_1_*c*_1_*r*_2_/*bc*_2_+*c*_1_*r*_2_)=8.519876 × 10^−6^.

The coexistence equilibrium point with parameters given in Table 4 is obtained as *x*^*∗*^=4.99 and *y*^*∗*^=5.99. At this point the patient can survive for some time period.

**Figure 11:**
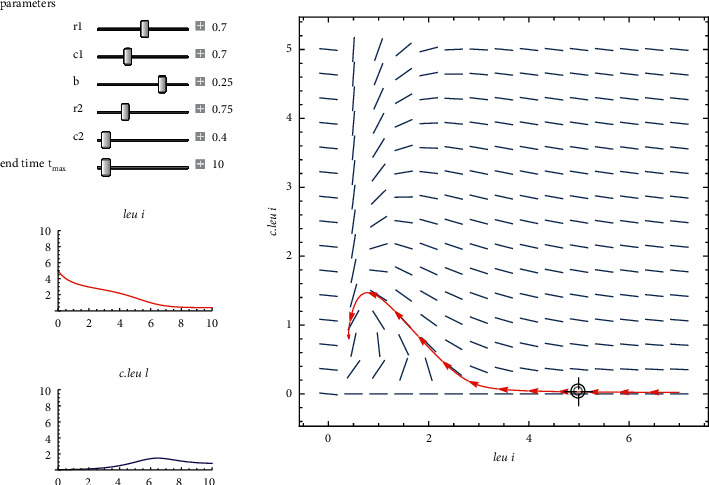
Leslie's predator-prey phase plane depicts the onset of the disease or the decrease in leukocytes following an increase in cancerous leukocytes.

Also, from Figure 11 if ${\left(\sqrt{r_{1}}-\sqrt{r_{2}}\right)}^{2}\geq \left(r_{1}c_{1}r_{2}/bc_{2}+c_{1}r_{2}\right)$ with parameters in Table 4 it gives ${\left(\sqrt{r_{1}}-\sqrt{r_{2}}\right)}^{2}=8.623253\times 10^{-4}$, (*r*_1_*c*_1_*r*_2_/*bc*_2_+*c*_1_*r*_2_)=0.588 then both the eigenvalues are positive i.e., *λ*_1_+*λ*_2_=0.638, *λ*_1_*λ*_2_=0.525 so it is unstable knot. This situation describes an acute leukemia i.e., variation of parameters shows a drastic change in patients condition.

For a chronic leukemia patient, the parameters are given in Table 4 and the critical point is *x*^*∗*^=0.5 and *y*^*∗*^=0.5. As it can be observed in Figure 8, in this case, the patient condition does not change drastically but still, it is an unstable node due to the positive eigen values i.e., *λ*_1_+*λ*_2_=0.3, *λ*_1_*λ*_2_=0.36

In the last section, a three player game interaction is described after the *HSC* transplantation between human normal *leu*, *c*.*leu*, and *HSC*. It is shown by taking two coalitions i.e., favourable and unfavourable. Different results have been obtained to show the accuracy and applicability of replicator dynamics to this disease. The phase portrait Figure 10 for the first coalition between favourable and unfavourable cells depicts the rise in *leu*. Which means that chemotherapy is being successful in eliminating the *c*.*leu*, the transplant have been successful and patient will surely survive.

The portrait in Figure 9 indicates the increase in unfavourable cell coalitions. The second coalition indicates how badly it can affect the life of the patient because the amount of cancerous leukocytes are limited at the time of chemotherapy, but if graft versus host disease happens after transplantation, it can destroy all the remaining leukocytes that cause the patient to die.

## 7. Conclusion & Future Work

This paper concludes that even a complex disease such as leukemia can be described using population dynamics and evolutionary game theory. These techniques have been implemented and their results provided. These models successfully deduce steady states and their stability. These models individually permit the existence of two types of stationary states:

1. State of no disease, with no cancerous leukocytes
2. State of coexistence where a cancerous leukocyte persists against the background of the immune response

The state of no disease is asymptotically stable and a state of leukemia is unstable. Both models are in complete agreement about stable and unstable situations. It was found from the study, that the state of no disease represents the immune state.

In this research, a two person zero-sum game of evolutionary game theory is used to describe the patient's condition having leukemia. Different results have been obtained for this situation by incorporating a mathematical software Mathematica. Then leukemia dynamics is studied by an ecological model Lesli predator prey model. Different results have also been obtained for this situation as well by incorporating a mathematical software Mathematica. The treatment of leukemia i.e., HSC is then studied by using a three player zero sum game as it was observed that evolutionary game theory gives a better understanding of leukemia than the ecological model. This study is a sincere effort to show another perspective of game theory that it can also define the complex dynamics of leukemia. By adding more complex factors, these calculations can give more realistic and accurate results.

The game is based on the number of leukocytes in this paper, but in future work, we may investigate evolutionary game theory for leukemia into specific cell types and their count to better determine which cell types should specifically be targeted and can result in eradicating the disease. Because current treatment advancements remove both healthy and malignant cells from the body, patients suffer greatly and are more susceptible to getting infected by a virus.

## Figures and Tables

**Table 1:** General form of the payoff matrix for cell to cell interaction.

	*c*.*l*(*c*.*leu*)	*I*(*leu*)

*c*.*l*(*c*.*leu*)	0	*a*_11_ − *a*_21_=−*a*

*I*(*leu*)	*a*_22_ − *a*_12_=*b*	0

**Table 2:** General form of the payoff matrix for cell/cell interaction in *HSC* transplant (coalition (*i*)).

	*F*_1_	*F*_2_

*F*_1_	0	*a* − *c*=−*a*_1_
*F*_2_	*d* − *b*=*a*_2_	0

**Table 3:** General form of the payoff matrix for cell/cell interaction in *HSC* transplant (coalition (*ii*)).

	*F*_1_	*F*_2_

*F*_1_	0	*e* − *g*=−*b*_1_
*F*_2_	*h* − *f*=*b*_2_	0

**Table 4:** Description of parameters for leukemia patient for different stages of disease.

Description	Early prognosis value	Acute leukemia	Chronic leukemia

*r*_1_	0.5	0.7	0.6
*c*_1_	1.42 × 10^−6^	0.7	0.6
*b*	0.1	0.25	0.6
*r*_2_	0.6	0.75	0.6
*c*_2_	0.5	0.4	0.6

## Data Availability

No data were used to support this study.
